# Expression and subcellular localization of kinetoplast-associated proteins in the different developmental stages of *Trypanosoma cruzi*

**DOI:** 10.1186/1471-2180-9-120

**Published:** 2009-06-04

**Authors:** Danielle Pereira Cavalcanti, Márcia Kiyoe Shimada, Christian Macagnan Probst, Thais Cristina Baeta Soares Souto-Padrón, Wanderley de Souza, Samuel Goldenberg, Stênio Perdigão Fragoso, Maria Cristina Machado Motta

**Affiliations:** 1 Laboratório de Ultraestrutura Celular Hertha Meyer, Instituto de Biofísica Carlos Chagas Filho, Universidade Federal do Rio de Janeiro, Rio de Janeiro, Brazil; 2 Instituto Nacional de Metrologia, Normalização e Qualidade Industrial, Inmetro, Rio de Janeiro, RJ, Brazil; 3 Instituto Carlos Chagas, Fiocruz, Curitiba, PR, Brazil; 4 Instituto de Microbiologia Prof. Paulo de Góes, Universidade Federal do Rio de Janeiro, Rio de Janeiro, Brazil

## Abstract

**Background:**

The kinetoplast DNA (kDNA) of trypanosomatids consists of an unusual arrangement of circular molecules catenated into a single network. The diameter of the isolated kDNA network is similar to that of the entire cell. However, within the kinetoplast matrix, the kDNA is highly condensed. Studies in *Crithidia fasciculata *showed that kinetoplast-associated proteins (KAPs) are capable of condensing the kDNA network. However, little is known about the KAPs of *Trypanosoma cruzi*, a parasitic protozoon that shows distinct patterns of kDNA condensation during their complex morphogenetic development. In epimastigotes and amastigotes (replicating forms) the kDNA fibers are tightly packed into a disk-shaped kinetoplast, whereas trypomastigotes (non-replicating) present a more relaxed kDNA organization contained within a rounded structure. It is still unclear how the compact kinetoplast disk of epimastigotes is converted into a globular structure in the infective trypomastigotes.

**Results:**

In this work, we have analyzed KAP coding genes in trypanosomatid genomes and cloned and expressed two kinetoplast-associated proteins in *T. cruzi*: TcKAP4 and TcKAP6. Such small basic proteins are expressed in all developmental stages of the parasite, although present a differential distribution within the kinetoplasts of epimastigote, amastigote and trypomastigote forms.

**Conclusion:**

Several features of TcKAPs, such as their small size, basic nature and similarity with KAPs of *C. fasciculata*, are consistent with a role in DNA charge neutralization and condensation. Additionally, the differential distribution of KAPs in the kinetoplasts of distinct developmental stages of the parasite, indicate that the kDNA rearrangement that takes place during the *T. cruzi *differentiation process is accompanied by TcKAPs redistribution.

## Background

Eukaryotic genomes are packaged into the nucleus by histones and non histone proteins. Histones are small, highly basic proteins that form a core around which the DNA is wrapped. Although chromatin is highly compacted, its structure is dynamic, allowing access to the DNA for processes such as replication, transcription, recombination and repair [1,2]. Nucleoid-associated proteins have been described in archaea and bacteria. These proteins resemble eukaryotic histones in their DNA binding properties, low molecular weight, abundance and electrostatic charge. They organize and compact the prokaryotic genome and are involved in various processes, including gene expression [3,4].

The proteins involved in DNA packaging in eukaryotic organelles have not been fully characterized. In the protozoa of the Trypanosomatidae family, the mitochondrial genome is contained within a specific region of the mitochondrion known as the kinetoplast. The kinetoplast DNA (kDNA) of trypanosomatids is organized into an unusual arrangement of circular molecules, catenated into a single network. Two types of DNA ring are present within the kinetoplast: maxicircles and minicircles. The maxicircles resemble the mitochondrial DNA of higher eukaryotes, encoding rRNAs and subunits of the respiratory complexes [5]. The minicircles encode guide RNAs, which modify the maxicircle transcripts by extensive insertions and/or deletions of uridylate residues to form functional open reading frames, in a process known as RNA editing [6]. The replication of kinetoplast DNA requires a repertoire of molecules, including type II topoisomerases, DNA polymerases, universal minicircle sequence binding proteins, primases and ribonucleases [7,8].

The molecules involved in maintaining the highly ordered organization of kDNA in trypanosomatids remained unknown for many years. In 1965, Steinert suggested that the kinetoplast DNA was not associated with basic proteins [9]. However, Souto-Padrón and De Souza provided cytochemical evidence that the kDNA of *Trypanosoma cruzi *was associated with basic proteins [10,11]. They suggested that such proteins might be involved in neutralizing the negatively charged DNA molecules in close contact within the kinetoplast matrix. The proteins involved in kDNA organization were not biochemically or molecularly characterized until the 1990s, following the isolation of kinetoplast-associated proteins (KAPs) from *Crithidia fasciculata *[12]. The KAPs of *C. fasciculata *characterized to date (CfKAP1, 2, 3 and 4) are small, highly basic proteins with a composition similar to that of the H1 histone, which contains lysine- and alanine-rich domains. These CfKAPs have a cleavable nine-amino acid presequence in their N-terminal region that is absent from mature forms and probably involved in kinetoplast import [13]. CfKAPs have been shown to be exclusively restricted to the kinetoplast in immunolocalization assays and to bind to minicircles and condense the kDNA network *in vitro *[13,14]. Several roles have been attributed to KAPs in *C. fasciculata*. They may facilitate the side-to-side association of individual strands of DNA through charge neutralization and influence the orientation of the kDNA to facilitate interaction with specific minicircle sequences [12-14]. Further evidence of the involvement of KAPs in kDNA organization *in vivo *was obtained by disrupting both alleles of the *KAP1 *gene of *C. fasciculata*. The double-knockout mutant was viable, but presented substantial kDNA rearrangement, including a high level of kDNA fiber packaging and the appearance of a thicker layer in the middle of the kinetoplast disk [15]. Surprisingly, this phenotypic modification was found to resemble the effects of treating *C. fasciculata *with topoisomerase II inhibitors [16]. The inability of KAPs 2, 3 and 4 to complement KAP1 function in kDNA organization is consistent with KAP1 having a role different from that of other KAPs. Indeed, KAP 2 and 3 are involved in mitochondrial metabolism rather than kDNA organization, as disruption of both alleles of the KAP 2 and 3 genes increases the levels of several mitochondrial mRNAs, reduces respiration rate and interferes with cell growth and morphology [17].

Despite some efforts to identify kinetoplast-associated proteins in *T. cruzi*, little is known about KAPs in this protozoon [18,19]. *T. cruzi *is the etiologic agent of Chagas disease and passes through several developmental stages during its life cycle. Epimastigotes and amastigotes are the proliferative forms found in the insect host midgut and mammalian cells, respectively, whereas trypomastigotes are the non proliferative forms infecting the vertebrate host [20]. The differentiation of epimastigotes into trypomastigotes involves morphological changes, including kDNA rearrangement. In the epimastigote and amastigote forms of *T. cruzi*, as in most trypanosomatids, the kDNA fibers are tightly packed into a compact disk-shaped structure. Conversely, trypomastigotes have a rounded kinetoplast, with a more relaxed organization of kDNA [21]. The conversion of the kinetoplast disk into a globular structure probably involves a mechanism controlling the type of KAPs associated with the kDNA or the extent to which these proteins associate with the DNA network at different stages of parasite development.

In this work, we have analyzed KAP coding sequences in trypanosomatid genomes, organized them by means of phylogenetic and syntenic analyses and defined two KAP proteins distinct from those originally described in *C. fasciculata*. In addition, two kinetoplast-associated proteins of *T*. cruzi, TcKAP4 and TcKAP6, were cloned, expressed and antisera were generated against recombinant proteins. Imunolabeling assays revealed a differential distribution of TcKAPs in the kinetoplast of distinct developmental stages of the parasite.

## Methods

### Cell culture

Epimastigote forms of *T. cruzi *(Dm28c clone) [22] were grown in liver infusion tryptose (LIT) medium supplemented with 10% fetal calf serum at 28°C. Bloodstream trypomastigote forms derived from the blood of Swiss mice were used to infect the LLC-MK2 cells. Trypomastigotes were released seven days after infection in the supernatant and purified by centrifugation. Amastigotes were obtained by disruption of the LLC-MK2 cells after four days of infection with trypomastigotes. It is worth mentioning that the amastigotes released after disruption of the cells are mixed with intermediate forms, which represent a transitional stage between amastigotes and trypomastigotes [20].

### DNA extraction

DNA was extracted as described by Medina-Acosta and Cross [23].

### Genome search for *T. cruzi *orthologs of CfKAPs

The CfKAPs1–4 protein sequences were retrieved from GenBank^® ^[24] and a BLASTp search [25] was performed against all protein sequences from trypanosomatids with a complete sequenced genome, available in GenBank^® ^(release 169). All hits having an e-value lower than 1e10^-5 ^were selected for further analyses. Sequences that were redundant or did not contain a discernible nine amino acids presequence, suggestive of kinetoplast import, were discarded.

### Evolutionary analysis of trypanosomatids KAPs

Multiple sequence alignments (MSAs) were produced with the ClustalW software [26] and a phylogenetic analysis was performed using the MrBayes software [27,28], running in parallel [29] in a 28 nodes cluster, by 20,000,000 generations, with gamma correction (estimated α = 6.675), allowing for invariant sites. A mixed amino acid model was used and the Wag fixed rate model [30] prevailed with a posterior probability of 1.0. MSAs and trees were visualized with the Jalview [31] and TreeView software [32], respectively

### Cloning and expression of the *TcKAP4 *and *TcKAP6 *genes

Primers were designed to amplify the entire coding region of these genes from the *T. cruzi *Dm28c genome. Primers TcKAP4-F (from nucleotide 1 to 29) (5' GGG**GGATCC**ATGCTCCGCTTTTGTCGGTCTCGCCTCGC 3') and TcKAP4-R (from nucleotide 356 to 384) (5' GGG**GTCGAC**TTAAGTCTTTTTCTTCTTTGGCTGCTGCT 3') were constructed based on the sequence with GenBank ID XM_801968 and used to amplify the *TcKAP4 *coding region, whereas the sequence with GenBank ID XM_810849 was used to design the primers TcKAP6-F (from nucleotide 1 to 21) (5' GCG**GGATCC**ATGCTTCGCGTCTCCCTGCTT 3') and TcKAP6-R (from nucleotide 531 to 558) (5' GGG**GTCGAC**TCACACCTTGCTACGTGTGATCTTCTTG 3') for PCR amplification of the *TcKAP6 *coding region. The forward primers contain *Bam*HI sites whereas the reverse primers contain *Sal*I sites (bold sequences). PCR was carried out in the following reaction mixture: 10 pmol of each pair of primers, 100 ng of *T. cruzi *genomic DNA, 200 μM dNTPs, 1.5 mM MgCl_2_, 20 mM Tris-HCl, pH 8.4, 50 mM KCl and 2.5 units of *Taq *DNA polymerase (Invitrogen). Reactions were carried out in a GeneAmp PCR System 9700 (Applied Biosystems) thermal cycler, with an initial denaturation at 94°C for 4 min, followed by 30 cycles of 94°C for 30 s, 58°C for 30 s and 72°C for 30 s. We obtained an amplified product of 0.4 kb for *TcKAP4 *and 0.65 kb for *TcKAP6*. The PCR products were purified with a high-purity PCR product purification kit (Roche), digested with *Bam*HI and *Sal*I and inserted into the pQE30 expression vector (QIAGEN). The His_6_-tagged recombinant proteins were produced in the *E. coli *M15 strain following induction with 1 mM IPTG (isopropyl-1-thio-β-D-galactopyranoside) and culture for an additional 3 h at 37°C.

### Purification of recombinant TcKAPs

The recombinant proteins were largely insoluble and were obtained from the inclusion bodies. The pellets of cultures of *E. coli *expressing *TcKAP4 *or *TcKAP6 *(250 ml) were resuspended in 10 ml of 20 mM Tris HCl, pH 8.0, 0.5 M NaCl and subjected to five pulses of sonication for 10 s each at 4°C (Cole Parmer 4710). The sonicated extracts of *E. coli *were centrifuged at 12,000 × *g *for 10 min at 4°C. The supernatant was discarded and the pellets containing the inclusion bodies were washed three times in 50 mM Tris-HCl, pH 8.0, 0.5 M NaCl, 2% Triton X-100, resuspended in 4 ml of the protein sample buffer for SDS-PAGE (Sodium Dodecyl Sulfate Polyacrylamide Gel Electrophoresis) and resolved in 15% polyacrylamide gels (20 cm × 20 cm × 0.4 cm) at 20 volts for 16 h at room temperature. After electrophoresis, the gels were incubated in cold KCl (100 mM) for 30 min to visualize the bands of proteins. The recombinant protein bands were excised from the gels, electroeluted in a dialysis bag at 60 V for 2 h in SDS-PAGE buffer and dialyzed against PBS (10 mM sodium phosphate buffer, 150 mM NaCl), pH 8.0.

### Production of polyclonal antisera

Polyclonal antisera against the recombinant proteins were produced in mice. The animals were immunized by intraperitoneal injection with 100 μg of the appropriate antigen in Freund's complete adjuvant (Sigma) for the first inoculation and with 20 μg of the recombinant protein with Freund's incomplete adjuvant (Sigma) for three booster injections at two-week intervals. Antisera were obtained five days after the last booster injection.

### Immunoblotting

For immunoblotting analysis, cell lysates (1 × 10^7 ^parasites) were separated by SDS-PAGE in 15% polyacrylamide gels and the protein bands were transferred onto a nitrocellulose membrane (Hybond C, Amersham Biosciences) according to standard protocols [33]. Nonspecific binding sites were blocked by incubating the membrane with 5% nonfat milk powder and 0.1% Tween-20 in PBS, pH 8.0 for 30 min. Membranes were then incubated for 1 h with the polyclonal antiserum raised against the recombinant protein (TcKAP4 or TcKAP6) diluted 1:500 in blocking solution. The membrane was washed three times in PBS and then incubated for 45 min with alkaline phosphatase-conjugated anti-mouse IgG secondary antibody (Sigma) diluted 1:10,000 in blocking solution. Bound antibodies were detected with the BCIP (5-bromo-4-chloro-3-indolyl-phosphate)/NBT (nitro blue tetrazolium) solution kit (Promega). The pre immune sera were also tested, as described above. The antibody anti-polyhistidine (Sigma) was diluted 1:3,000 in blocking solution and used to confirm the expression of TcKAPs in *E. coli *M15 strain.

### Immunofluorescence assays

The parasites were washed in PBS, pH 8.0 and fixed by incubation with 4% freshly prepared formaldehyde in PBS for 30 min. Cells were deposited on poly-L-lysine-treated microscope slides and permeabilized by incubation with 0.5% Triton X-100 in PBS, pH 8.0, for 5 min. The slides were incubated in blocking solution containing 1.5% BSA, 0.5% teleostean gelatin, 0.02% Tween 20 in PBS, pH 8.0 and were then incubated with anti-TcKAP4 or anti-TcKAP6 antiserum diluted 1:80 in blocking solution for 1 h. The parasites were washed and incubated with Alexa Fluor^® ^488 goat anti-mouse IgG (Molecular Probes) diluted 1:500 in blocking solution for 45 min. The pre immune sera were also tested, as described above. The slides were mounted in N-propyl gallate and visualized by confocal laser scanning microscope (Zeiss LSM510 META). For control assays, the incubation with anti-TcKAP4 or anti-TcKAP6 was omitted.

### Transmission electron microscopy

Protozoa were fixed in 2.5% glutaraldehyde diluted in 0.1 M cacodylate buffer, pH 7.2, for 2 h at room temperature and post-fixed in 0.1 M cacodylate buffer containing 1% OsO_4_, 5 mM calcium chloride and 0.8% potassium ferricyanide for 1 h. Then, cells were dehydrated in a graded series of acetone and embedded in Epoxy resin. Ultrathin sections were stained with uranyl acetate and lead citrate and observed in a Zeiss 900 transmission electron microscope.

### Ultrastructural immunocytochemistry

The parasites were fixed in 0.3% glutaraldehyde, 4% formaldehyde and 1% picric acid diluted in 0.1 M cacodylate buffer, pH 7.2 and then dehydrated at -20°C in a graded series of ethanol solutions. The material was progressively infiltrated with Unicryl at lower temperatures and resin polymerization was carried out in BEEM capsules at -20°C for 5 days, under ultraviolet light. Ultrathin sections were obtained with a Leica ultramicrotome (Reichert Ultracuts) and grids containing the sections were incubated with 50 mM NH_4_Cl for 30 min. They were then incubated with blocking solution (3% BSA, 0.5% teleostean gelatin diluted in PBS, pH 8.0) for 30 min, followed by incubation with anti-TcKAP4 or anti-TcKAP6 antiserum diluted 1:100 in blocking solution for 1 h. The grids were then incubated for 30 min with gold-labeled goat anti-mouse IgG (Sigma) diluted 1:200 in blocking solution, washed and stained with uranyl acetate and lead citrate for further observation in a Zeiss 900 transmission electron microscope. For control assays, incubation with the primary antiserum was omitted.

## Results and discussion

### Bioinformatic analysis of kinetoplast-associated proteins in trypanosomatid species

**Figure 1:**
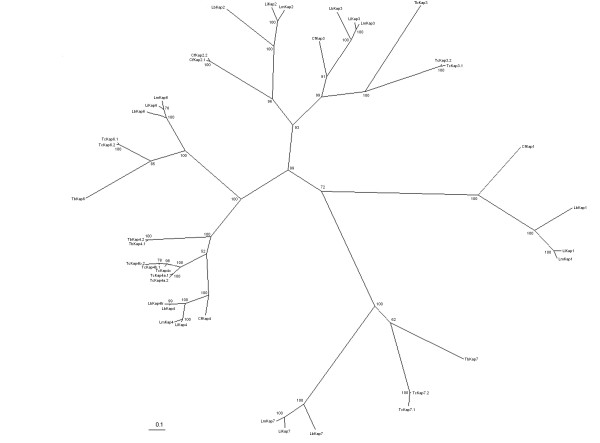
**Phylogenetic analysis of trypanosomatid KAPs proteins with confidence values shown as percentages**. Lb, *Leishmania braziliensis; *Li, *Leishmania infantum; *Lm, *Leishmania major*; Tb, *Trypanosoma brucei*; Tc, *Trypanosoma cruzi*.

As previously stated, originally five distinct kinetoplast-associated proteins were described in *C. fasciculata*, named CfKAP1–5 [12,13]. However, the CfKAP5, also designated p15, was never characterized. Since little is known about kDNA-associated proteins in *T. cruzi *[18,19] and other trypanosomatids, we sought initially to address this problem by examining genome database of the *T. cruzi *[34], *T. brucei *[35], *Leishmania major *[36], *L. infantum *and *L. braziliensis *[37]. In a BLASTp search, using as query the available CfKAP protein sequences, we have identified 35 protein sequences related to CfKAPs: 11 in *T. cruzi; *7 in *L. braziliensis; *6 in *L. major *and *L. infantum; *and 5 in *T. brucei*. A phylogenetic analysis including these 35 sequences and the five CfKAPs used as query was performed, in order to construct a phylogenetic tree (figure 1). Additionally, a synteny conservation analysis was performed, where chromosome location was highly correlated with tree topology, allowing us to infer the homology relationships between the trypanosomatid KAPs [see additional file 1].

In the *T. cruzi *genome, we were able to identify the KAP3 and KAP4 genes, but not the KAP1 and KAP2 genes, which were only identified in *Leishmania *spp. and *C. fasciculata*. Furthermore, we were able to identify two other genes that are similar to the CfKAPs, herein named KAP6 and KAP7. They have not been characterized in *Crithidia*, as the available sequence information for this genome is limited (227 nucleotide sequences in the current version of GenBank). The KAP6 gene whose size is compatible to others KAPs, is more related to KAP4 (figure 1) and was annotated in all five genomes analyzed as "kinetoplast DNA-associated protein". The KAP7 gene, also present in all trypanosomatid genomes, has been annotated as "hypothetical protein, conserved". Although it is clustered with the KAP1 gene (figure 1), the lower bootstrap value of this clade reinforces the uncertainty of KAP7 relationship to other KAPs. The KAP genes of *T. cruzi *are present as two copies, with the exception of TcKAP4c, probably due to the hybrid nature of the CL Brener strain [34]- [see additional file 1].

### Characterization of TcKAPs

**Figure 2:**
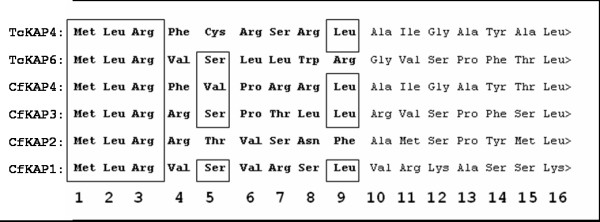
**Comparison of N-terminal sequences of KAPs from *C. fasciculata *and *T. cruzi***. The presequences predicted to be involved in kinetoplast import are shown in bold type. The boxes indicate the highly conserved amino acids. Note that all sequences begin with the sequence M, L, R. In all sequences other than those of CfKAP4 and TcKAP4, the fifth amino acid is hydroxylated and the ninth is generally hydrophobic. CfKAP4 (PIR JC6092), CfKAP3 (GenBank accession number AY143553), CfKAP2 (GenBank accession numbers AF008943 and AF008944) and CfKAP1 (GenBank accession number AF034951) are KAPs from *C. fasciculata *whereas TcKAP4 (GenBank accession number ABR15473) and TcKAP6 (GenBank accession number ABR15474) are *T. cruzi *KAPs.

In this work, we cloned and expressed two KAPs in *T. cruzi*: TcKAP4 and TcKAP6. The PCR amplification of the TcKAP4a gene generated a 387 bp DNA fragment encoding a protein with a predicted molecular weight of 14.5 kDa. The deduced amino acid sequence of the protein encoded by the TcKAP4 gene includes 28% basic residues, with a predicted pI of 14.5. The TcKAP6 gene is 558 base pairs long and encodes a polypeptide with a predicted molecular weight of 21.2 kDa. The amino acid sequence of TcKAP6 includes 30% of basic residues and this protein has a predicted pI of 11.3. The amino acid sequence data reported here are available from GenBank under the accession numbers ABR15473 for TcKAP4 and ABR15474 for TcKAP6. Both TcKAP4 and TcKAP6 have a clearly identifiable cleavable presequence in the N-terminal region similar to that described for the KAPs of *C. fasciculata *and potentially involved in mitochondrial import (figure 2). These presequences are absent from the mature forms of the proteins in *C. fasciculata *and with the exception of their length, have all the properties usually associated with cleavable mitochondrial presequences [12-14]. Similar sequences have been identified in the *C. fasciculata *kinetoplast DNA polymerase beta, *T. brucei *hsp60 and *Leishmania tarentolae *aldehyde dehydrogenase [38-40].

As reported for their counterparts in *C. fasciculata *[12,13], the TcKAPs are positively charged and small, consistent with a role in DNA charge neutralization and kDNA condensation in *T. cruzi*. The interaction between KAPs and kDNA may involve nonspecific electrostatic binding to DNA, interaction with specific regions of the minicircles or both types of association. However, further studies are required to investigate the occurrence of interaction between TcKAPs and kDNA, and how these interactions determine DNA network organization in *T. cruzi*.

### Detection of TcKAPs in the distinct developmental stages of *T. cruzi*

**Figure 3:**
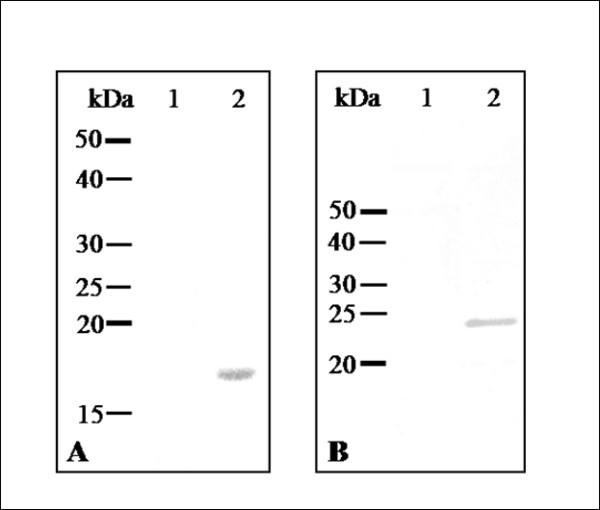
**Expression of recombinant TcKAPs in *E. coli***. The TcKAP4 (A) and TcKAP6 (B) were expressed in *E. coli *M15 strain following induction with 1 mM IPTG for 3 h. Immunoblotting assays of non-induced (1) and induced (2) bacterial extract using anti-polyhistidine antibody confirmed the expression of recombinant TcKAPs.

**Figure 4:**
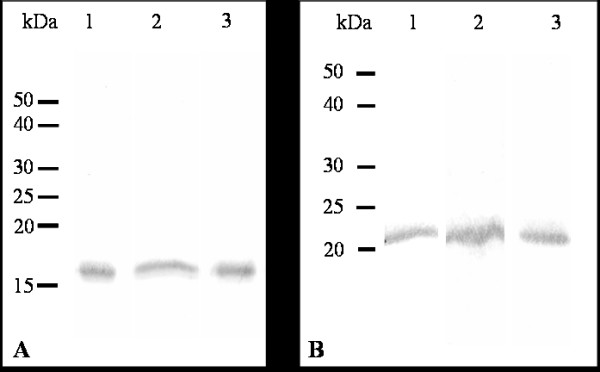
**Detection of TcKAPs in *T. cruzi***. Western blot analyses of (1) epimastigote, (2) amastigote/intermediate form and (3) trypomastigote extracts of *T. cruzi*, using anti-TcKAP4 (A) or anti-TcKAP6 (B) serum. Both antisera recognized a single polypeptide in all developmental stages of the parasite.

After cloning and expression, recombinant TcKap4 and TcKap6 proteins (figure 3) were purified in order to produce mouse polyclonal antisera against them. These antisera were used in immunoblotting assays, to analyze the expression of TCKAPs in proliferative and non proliferative stages of *T. cuzi*. Cell extracts of epimastigotes, amastigotes/intermediate forms and trypomastigotes were used and both antisera were able to detect a single polypeptide in all developmental stages of *T. cruzi*. The anti-TcKap4 serum detected a protein with an apparent molecular weight of 16 kDa (figure 4A) and the anti-TcKap6 serum detected a protein with an apparent molecular weight of 22 kDa (figure 4B). The molecular weights observed on SDS-PAGE were slightly higher than those expected based on the deduced amino acid sequences of TcKap4 and TcKap6. This difference may result from the basic nature of these proteins.

### The kinetoplast ultrastructure in *T. cruzi *and distribution of TcKAPs

**Figure 5:**
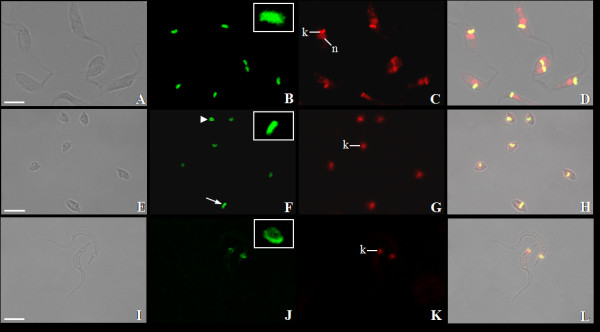
**Distribution of TcKAP4 in *T. cruzi***. Immunolocalization of TcKAP4 in epimastigotes (A-D), amastigotes/intermediate forms (E-H) and trypomastigotes (I-L) of *T. cruzi*. In epimastigotes (B) and amastigotes (F-arrow), the protein is distributed throughout the kDNA disk (insets). In intermediate forms (F-arrowhead) and trypomastigotes (J-inset), a peripheral labeling of the kinetoplast was observed. (A-E-I) Phase-contrast image, (B-F-J) fluorescence image using anti-TcKAP4 serum, (C-G-K) propidium iodide showing the nucleus (n) and the kinetoplast (k), and (D-H-L) the overlay image. Bars = 5 μm.

**Figure 6:**
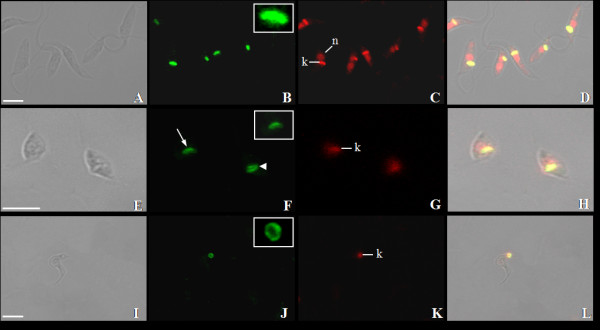
**Distribution of TcKAP6 in *T. cruzi***. Immunolocalization of TcKAP6 in epimastigotes (A-D), amastigotes/intermediates forms (E-H) and trypomastigotes (I-L) of *T. cruzi*. As observed for TcKAP4, this protein was also distributed throughout kDNA disk in epimastigotes (B-inset) and amastigotes (F-arrow and inset), and at the periphery of the kinetoplast in intermediate forms (F-arrowhead) and trypomastigotes (J-inset). (A-E-I) Phase-contrast image, (B-F-J) location of TcKAP6 in the kinetoplasts of *T. cruzi*, (C-G-K) iodide propidium labeling and (D-H-L) the overlay image. k = kinetoplast, n = nucleus. Bars = 5 μm.

**Figure 7:**
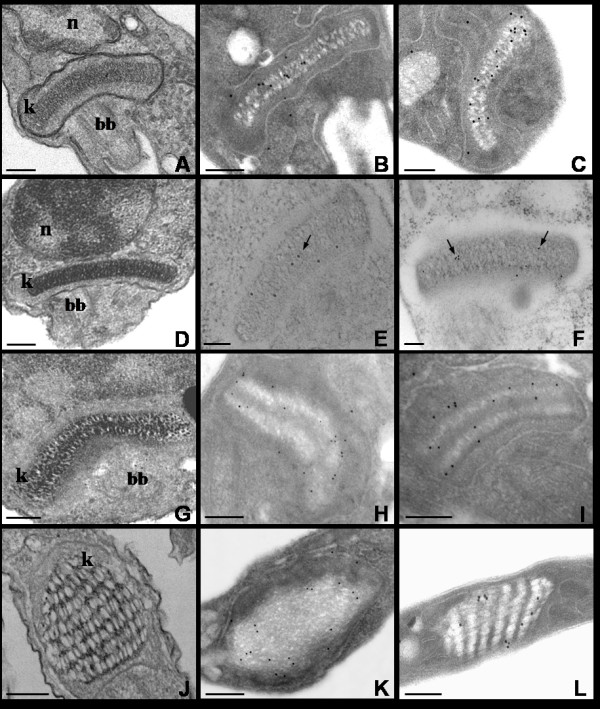
**Ultrastructural localization of TcKAPs by transmission electron microscopy**. A-D-G-J: ultrastructural analyses of the kinetoplast in the different developmental stages of *T*. cruzi. The kinetoplast of intermediate forms (G) is larger than the bar-shaped kinetoplast of epimastigotes (A) and amastigotes (D). The trypomastigotes (J) present a more relaxed kDNA organization, contained within a rounded kinetoplast. TcKAP4 (B-E-H-K) was distributed throughout the kinetoplast DNA network in epimatigotes (B) and amastigotes (E-arrow). In intermediate forms (H) and in trypomastigotes (K), TcKAP4 was distributed mainly at the periphery of the kDNA. The same result was observed for TcKAP6 (C-F-I-L). A homogenous distribution for all kinetoplast was observed in epimastigotes (C) and amastigotes (F-arrows), while a more peripherical distribution was seen in intermediate forms (I) and trypomastigotes (L). Bars = 0.25 μm. k = kinetoplast, n = nucleus, bb = basal body.

The TcKAP antisera were also employed to determine the subcellular location of TcKAPs in *T. cruzi*. It is worth mentioning that the kinetoplast of this parasite undergoes morphological changes during the protozoon life cycle; epimastigotes and amastigotes have tightly packed kDNA fibers condensed within the kinetoplast disk, whereas trypomastigotes have a more relaxed organization of kDNA, which is enclosed in a rounded structure. During the transformation of amastigotes in trypomastigotes inside the mammalian cell, changes occur in the general organization of the protozoa, in special in the kinetoplast structure. The population of intracellular parasites does not differentiate in perfect synchrony, thus at a certain time of the differentiation process, some transitional stages between amastigotes and trypomastigotes can be found in the same cell [20]. For this reason, the amastigotes used in our assays, which were released after disruption of LLC-MK2 cells, were mixed with intermediate forms. The kinetoplast of these intermediate forms is enlarged in relation to the disk observed in amastigotes, presenting the DNA fibers densely packed in the central area, but less condensed at the periphery. In order to analyze the distribution of TcKAPs in all developmental stages of *T. cruzi*, we carried out immunolabelling assays using TcKAP antisera in epimastigotes, amastigotes/intermediate forms and trypomastigotes. Both antisera specifically recognized the kinetoplast of all developmental stages of *T. cruzi *(figures 5 and 6). However, the distribution of these proteins within the kinetoplast depended on the developmental form of the parasite. In epimastigotas and amastigotes, TcKap4 and TcKap6 were distributed throughout the kDNA network (figures 5A–H for TcKAP4 and 6A–H for TcKAP6), consistent with findings for *C. fasciculata*, in which CfKAPs are distributed for all kinetoplast disk [13,14]. In intermediate forms (figures 5F and 6F, arrowheads) and trypomastigotes (figures 5 and 6I–L), TcKap4 and TcKap6 were distributed mainly at the periphery of the kDNA network. In order to better understand the kDNA arrangement present in the intermediate forms and the distribution of KAPs in the different developmental stages of *T. cruzi*, ultrastructural analyses and immunocytochemistry assays were performed (figure 7). In epimastigotes and amastigotes (figure 7A and 7D, respectively), which present a disk-shaped kinetoplast, we could observe gold particles distributed throughout the kinetoplast disk when both antisera were used (figure 7B and 7E for TcKAP4 and 7C and 7F for TcKAP6). In intermediate forms, which present an enlarged kinetoplast when compared to the disk-shaped kinetoplast of amastigotes (figure 7G), labeling of TcKAPs is more intense at the peripheral region than in the central area (figure 7H and 7I). In trypomastigotes, which present a round-shaped kinetoplast (figure 7J), gold particles were mainly observed at the periphery of the kinetoplast network (figure 7K and 7L), confirming the results obtained by immunofluorescence analysis. Preliminary cytochemical studies had already shown different distributions of basic proteins in the kinetoplasts of the different developmental stages of *T. cruzi *[41]. However, the reason for this differential protein distribution remain unclear. It is possible that these basic proteins are involved in topological rearrangements of the kDNA network during the *T. cruzi *life cycle, in which the compact bar-shaped kinetoplast is converted into a globular structure. However, no data are currently available to confirm or refute this hypothesis.

In this work we showed for the first time that the distribution of TcKAPs in different developmental stages of *T. cruzi *is related to the kinetoplast format: in disk-shaped structures, like those found in epimastigotes and amastigotes, proteins are seen dispersed through the kDNA network. Conversely, in intermediate and rounded kinetoplasts, like those observed in intermediate forms and trypomastigotes, KAPs are mainly located at the kDNA periphery. Taken together, these data indicate that the kDNA rearrangement that takes place during the *T. cruzi *differentiation process, is accompanied by TcKAP4 and TcKAP6 redistribution within the kinetoplast. It means that TcKAPs could determine, at least in part, the distinct topological organization of the kDNA networks.

Although much information is available concerning the kinetoplast-associated proteins in *C. fasciculata*, it is still unknown how KAPs and other proteins interact with the DNA molecules to condense and determine the tridimensional arrangement of the kDNA network in trypanosomatids. Further studies using gene knockout to inhibit the expression of KAPs or assays to over-express these proteins, would help us understand the biological function of TcKAPs in *T. cruzi *and their involvement (or not) in the topological rearrangements of kDNA during the parasite morphogenetic development.

## Conclusion

TcKAPs are candidate proteins for kDNA packaging and organization in *T. cruzi*. The trypanosomatid genomes sequenced to date have several sequences that share some degree of similarity with CfKAPs studied so far (CfKAP1–4). We have organized these sequences according to coding and syntenic information and have identified two potentially novel KAPs in these organisms, KAP6 and KAP7. Additionally, we have characterized two KAPs in *T. cruzi*, TcKAP4 and TcKAP6, which are small and basic proteins that are expressed in proliferative and non-proliferative stages of the parasite. TcKAPs present differential distribution within the kinetoplasts of epimastigotes, amastigotes, intermediate forms and trypomastigotes, indicating that the kDNA rearrangement that takes place during the *T. cruzi *differentiation process is accompanied by TcKAPs redistribution.

## Abbreviations

KAPs: kinetoplast-associated proteins; CfKAPs: kinetoplast-associated proteins of *C. fasciculata*; TcKAPs: kinetoplast-associated proteins of *T. cruzi*

## Authors' contributions

DPC carried out the experiments and wrote the manuscript. MKS helped to produce the mouse polyclonal antisera. CMP performed the phylogenetic and bioinformatic analysis. TCBSSP provided amastigotes and helped to analyze the results of the imunolabeling assays. WS and SG helped to analyze the results and revised the manuscript. SPF participated in the design and coordination of the study and helped to revise the manuscript. MCMM conceived the study and critically analyzed the paper content. All authors read and approved the final manuscript.

## Supplementary Material

Additional file 1 **Bioinformatic analysis of kinetoplast-associated proteins in trypanosomatid species**. These data provide a detailed bioinformatic analysis of kinetoplast-associated proteins in trypanosomatids, including: KAPs genome localization, alignment of the KAP genes and a table containing KAPs genebank ID. Click here for file

## References

[B1] Kornberg RD Lorch Y Twenty-five years of the nucleosome, fundamental particle of the eukaryote chromosome Cell 1999 98 285 294 10458604 10.1016/s0092-8674(00)81958-3

[B2] Polo SE Almouzni G Chromatin assembly: a basic recipe with various flavours Curr Opin Genet Dev 2006 16 104 111 16504499 10.1016/j.gde.2006.02.011

[B3] Sandman K Reeve JN Archaeal chromatin proteins: different structures but common function? Curr Opin Microbiol 2005 8 656 661 16256418 10.1016/j.mib.2005.10.007

[B4] Luijsterburg MS Noom MC Wuite GJ Dame RT The architectural role of nucleoid-associated proteins in the organization of bacterial chromatin: a molecular perspective J Struct Biol 2006 156 262 272 16879983 10.1016/j.jsb.2006.05.006

[B5] Shapiro TA Englund PT The structure and replication of kinetoplast DNA Annu Rev Microbiol 1995 49 117 43 8561456 8561456 10.1146/annurev.mi.49.100195.001001

[B6] Stuart K Panigrahi AK RNA editing: complexity and complications Mol Microbiol 2002 45 591 596 12139607 10.1046/j.1365-2958.2002.03028.x

[B7] Shlomai J The structure and replication of kinetoplast DNA Curr Mol Med 2004 4 623 647 15357213 10.2174/1566524043360096

[B8] Liu B Liu Y Motyka SA Agbo EEC Englund PT Fellowship of the rings: the replication of kinetoplast DNA Trends Parasitol 2005 21 363 369 15967722 10.1016/j.pt.2005.06.008

[B9] Steinert M L'absence d'histone dans le kinétonucleus des trypanosomes Exp Cell Res 1965 39 69 72 5831251 10.1016/0014-4827(65)90008-x

[B10] Souto-Padrón T De Souza W Ultrastructural localization of basic proteins in *Trypanosoma cruzi* J Histochem Cytochem 1978 26 349 358 77871 10.1177/26.5.77871

[B11] Souto-Padrón T De Souza W Cytochemical analysis at the fine-structural level of trypanosomatids stained with phosphotungstic acid J Protozool 1979 26 551 557 94607 10.1111/j.1550-7408.1979.tb04194.x

[B12] Xu C Ray DS Isolation of proteins associated with kinetoplast DNA networks *in vivo* Proc Natl Acad Sci USA 1993 90 1786 1789 8446592 10.1073/pnas.90.5.1786 PMC45964

[B13] Xu CW Hines JC Engel ML Russel DG Ray DS Nucleus-encoded histone H1-like proteins are associated with kinetoplast DNA in the trypanosomatid *Crithidia fasciculata* Mol Cell Biol 1996 16 564 576 8552084 10.1128/mcb.16.2.564 PMC231035

[B14] Hines JC Ray DS The *Crithidia fasciculata *KAP1 gene encodes a highly basic protein associated with kinetoplast DNA Mol Biochem Parasitol 1998 94 41 52 9719509 10.1016/s0166-6851(98)00048-6

[B15] Lukes J Hines JC Evans CJ Avliyakulov NK Prabhu VP Chen J Ray DS Disruption of the *Crithidia fasciculata *KAP1 gene results in structural rearrangement of the kinetoplast disc Mol Biochem Parasitol 2001 117 179 186 11606228 10.1016/s0166-6851(01)00348-6

[B16] Cavalcanti DP Fragoso SP Goldenberg S De Souza W Motta MCM The effect of topoisomerase II inhibitors on the kinetoplast ultrastructure Parasitol Res 2004 94 439 448 15517387 10.1007/s00436-004-1223-4

[B17] Avliyakulov NK Lukes J Ray DS Mitochondrial histone-like DNA-binding proteins are essential for normal cell growth and mitochondrial function in *Crithidia fasciculata* Eukaryotic Cell 2004 3 518 526 15075280 10.1128/EC.3.2.518-526.2004 PMC387644

[B18] Zavala-Castro JE Acosta-Viana K Guzmán-Marín E Rosado-Barrera ME Rosales-Encina JL Stage specific kinetoplast DNA-binding proteins in *Trypanosoma cruzi* Acta Trop 2000 76 139 146 10936573 10.1016/s0001-706x(00)00079-6

[B19] González A Rosales JL Ley V Díaz C Cloning and characterization of a gene coding for a protein (KAP) associated with the kinetoplast of epimastigotes and amastigotes of *Trypanosoma cruzi* Mol Biochem Parasitol 1990 40 233 243 1694571 10.1016/0166-6851(90)90045-n

[B20] De Souza W From the cell biology to the development of new chemotherapeutic approaches against trypanosomatids: dreams and reality Kinetoplastid Biol Dis 2002 1 3 12234386 10.1186/1475-9292-1-3 PMC119324

[B21] De Souza W Cell biology of *Trypanosoma cruzi* Int Rev Cytol 1984 86 197 283 6368447 10.1016/s0074-7696(08)60180-1

[B22] Contreras VT Araujo-Jorge TC Bonaldo MC Thomaz N Barbosa HS Meirelles MN Goldenberg S Biological aspects of the Dm 28c clone of *Trypanosoma cruzi *after metacyclogenesis in chemically defined media Mem Inst Oswaldo Cruz 1988 83 123 133 3074237 10.1590/s0074-02761988000100016

[B23] Medina-Acosta E Cross GA Rapid isolation of DNA from trypanosomatid protozoa using a simple 'mini-prep' procedure Mol Biochem Parasitol 1993 59 327 329 8341329 10.1016/0166-6851(93)90231-l

[B24] Benson DA Karsch-Mizrachi I Lipman DJ Ostell J Wheeler DL GenBank Nucleic Acids Res 2008 36 D25 30 18073190 10.1093/nar/gkm929 PMC2238942

[B25] Altschul SF Gish W Miller W Myers EW Lipman DJ Basic local alignment search tool J Mol Biol 1990 215 403 410 2231712 10.1016/S0022-2836(05)80360-2

[B26] Larkin MA Blackshields G Brown NP Chenna R McGettigan PA McWilliam H Valentin F Wallace IM Wilm A Lopez R Thompson JD Gibson TJ Higgins DG ClustalW and ClustalX version 2 Bioinformatics 2007 23 2947 2948 17846036 10.1093/bioinformatics/btm404

[B27] Huelsenbeck JP Ronquist F MRBAYES: Bayesian inference of phylogeny Bioinformatics 2001 17 754 755 11524383 10.1093/bioinformatics/17.8.754

[B28] Ronquist F Huelsenbeck JP MRBAYES 3: Bayesian phylogenetic inference under mixed models Bioinformatics 2003 19 1572 1574 12912839 10.1093/bioinformatics/btg180

[B29] Altekar G Dwarkadas S Huelsenbeck JP Ronquist F Parallel Metropolis-coupled Markov chain Monte Carlo for Bayesian phylogenetic inference Bioinformatics 2004 20 407 415 14960467 10.1093/bioinformatics/btg427

[B30] Whelan S Goldman N A general empirical model of protein evolution derived from multiple protein families using a maximum-likelihood approach Mol Biol Evol 2001 18 5 691 9 11319253 11319253 10.1093/oxfordjournals.molbev.a003851

[B31] Clamp M Cuff J Searle SM Barton GJ The Jalview Java Alignment Editor Bioinformatics 2004 20 426 427 14960472 10.1093/bioinformatics/btg430

[B32] Page RDM TREEVIEW: An application to display phylogenetic trees on personal computers CABIOS 1996 12 357 358 8902363 10.1093/bioinformatics/12.4.357

[B33] Sambrook J Fritsch EF Maniatis T Molecular Cloning. A Laboratory Manual 1989 Cold Spring Harbor, New York

[B34] El-Sayed NM Myler PJ Bartholomeu DC Nilsson D Aggarwal G Tran AN Ghedin E Worthey EA Delcher AL Blandin G Westenberger SJ Caler E Cerqueira GC Branche C Haas B Anupama A Arner E Aslund L Attipoe P Bontempi E Bringaud F Burton P Cadag E Campbell DA Carrington M Crabtree J Darban H da Silveira JF de Jong P Edwards K Englund PT Fazelina G Feldblyum T Ferella M Frasch AC Gull K Horn D Hou L Huang Y Kindlund E Klingbeil M Kluge S Koo H Lacerda D Levin MJ Lorenzi H Louie T Machado CR McCulloch R McKenna A Mizuno Y Mottram JC Nelson S Ochaya S Osoegawa K Pai G Parsons M Pentony M Pettersson U Pop M Ramirez JL Rinta J Robertson L Salzberg SL Sanchez DO Seyler A Sharma R Shetty J Simpson AJ Sisk E Tammi MT Tarleton R Teixeira S Van Aken S Vogt C Ward PN Wickstead B Wortman J White O Fraser CM Stuart KD Andersson B The genome sequence of *Trypanosoma cruzi*, etiologic agent of Chagas disease Science 2005 309 409 415 16020725 10.1126/science.1112631

[B35] Berriman M Ghedin E Hertz-Fowler C Blandin G Renauld H Bartholomeu DC Lennard NJ Caler E Hamlin NE Haas B Böhme U Hannick L Aslett MA Shallom J Marcello L Hou L Wickstead B Alsmark UC Arrowsmith C Atkin RJ Barron AJ Bringaud F Brooks K Carrington M Cherevach I Chillingworth TJ Churcher C Clark LN Corton CH Cronin A Davies RM Doggett J Djikeng A Feldblyum T Field MC Fraser A Goodhead I Hance Z Harper D Harris BR Hauser H Hostetler J Ivens A Jagels K Johnson D Johnson J Jones K Kerhornou AX Koo H Larke N Landfear S Larkin C Leech V Line A Lord A Macleod A Mooney PJ Moule S Martin DM Morgan GW Mungall K Norbertczak H Ormond D Pai G Peacock CS Peterson J Quail MA Rabbinowitsch E Rajandream MA Reitter C Salzberg SL Sanders M Schobel S Sharp S Simmonds M Simpson AJ Tallon L Turner CM Tait A Tivey AR Van Aken S Walker D Wanless D Wang S White B White O Whitehead S Woodward J Wortman J Adams MD Embley TM Gull K Ullu E Barry JD Fairlamb AH Opperdoes F Barrell BG Donelson JE Hall N Fraser CM Melville SE El-Sayed NM The genome of the African trypanosome *Trypanosoma brucei* Science 2005 309 416 422 16020726 10.1126/science.1112642

[B36] Ivens AC Peacock CS Worthey EA Murphy L Aggarwal G Berriman M Sisk E Rajandream MA Adlem E Aert R Anupama A Apostolou Z Attipoe P Bason N Bauser C Beck A Beverley SM Bianchettin G Borzym K Bothe G Bruschi CV Collins M Cadag E Ciarloni L Clayton C Coulson RM Cronin A Cruz AK Davies RM De Gaudenzi J Dobson DE Duesterhoeft A Fazelina G Fosker N Frasch AC Fraser A Fuchs M Gabel C Goble A Goffeau A Harris D Hertz-Fowler C Hilbert H Horn D Huang Y Klages S Knights A Kube M Larke N Litvin L Lord A Louie T Marra M Masuy D Matthews K Michaeli S Mottram JC Müller-Auer S Munden H Nelson S Norbertczak H Oliver K O'neil S Pentony M Pohl TM Price C Purnelle B Quail MA Rabbinowitsch E Reinhardt R Rieger M Rinta J Robben J Robertson L Ruiz JC Rutter S Saunders D Schäfer M Schein J Schwartz DC Seeger K Seyler A Sharp S Shin H Sivam D Squares R Squares S Tosato V Vogt C Volckaert G Wambutt R Warren T Wedler H Woodward J Zhou S Zimmermann W Smith DF Blackwell JM Stuart KD Barrell B Myler PJ The genome of the kinetoplastid parasite *Leishmania major* Science 2005 309 436 442 16020728 10.1126/science.1112680 PMC1470643

[B37] Peacock CS Seeger K Harris D Murphy L Ruiz JC Quail MA Peters N Adlem E Tivey A Aslett M Kerhornou A Ivens A Fraser A Rajandream MA Carver T Norbertczak H Chillingworth T Hance Z Jagels K Moule S Ormond D Rutter S Squares R Whitehead S Rabbinowitsch E Arrowsmith C White B Thurston S Bringaud F Baldauf SL Faulconbridge A Jeffares D Depledge DP Oyola SO Hilley JD Brito LO Tosi LR Barrell B Cruz AK Mottram JC Smith DF Berriman M Comparative genomic analysis of three *Leishmania *species that cause diverse human disease Nat Genet 2007 39 839 847 17572675 10.1038/ng2053 PMC2592530

[B38] Bringaud F Peyruchaud S Baltz D Giroud C Simpson L Baltz T Molecular characterization of the mitochondrial heat shock protein 60 gene from *Trypanosoma brucei* Mol Biochem Parasitol 1995 74 119 123 8719252 10.1016/0166-6851(95)02486-7

[B39] Bringaud F Peris M Zen KH Simpson L Characterization of two nuclear-encoded protein components of mitochondrial ribonucleoprotein complexes from *Leishmania tarentolae* Mol Biochem Parasitol 1995 71 65 79 7630384 10.1016/0166-6851(95)00023-t

[B40] Torri AF Englund PT A DNA polymerase b in the mitochondrion of the trypanosomatid *Crithidia fasciculata* J Biol Chem 1995 270 8 3495 7 7876082 7876082 10.1074/jbc.270.8.3495

[B41] Esponda P Souto-Padrón T De Souza W Fine structure and cytochemistry of the nucleus and the kinetoplast of epimastigotes of *Trypanosoma cruzi* J Protozool 1983 30 105 110 6191027 10.1111/j.1550-7408.1983.tb01041.x

