# Supplementary material for: Expression and subcellular localization of kinetoplast-associated proteins in the different developmental stages of Trypanosoma cruzi
Source: BMC Microbiol. 2009 Jun 4;9:120. doi: 10.1186/1471-2180-9-120 (PMC2700280; doi:10.1186/1471-2180-9-120)
Supplement: Additional file 1 — Bioinformatic analysis of kinetoplast-associated proteins in trypanosomatid species. These data provide a detailed bioinformatic analysis of kinetoplast-associated proteins in trypanosomatids, including: KAPs genome localization, alignment of the KAP genes and a table containing KAPs genebank ID. [file 1471-2180-9-120-S1.doc]

**ADDITIONAL FILE**

**Bioinformatic analysis of kinetoplast-associated proteins in trypanosomatid species**

The bulk of knowledge about the packaging, maintenance and replication of the kDNA network has come from studies of *C. fasciculata* and *T. brucei*. Little is known about the proteins associated with the kDNA of *T. cruzi*. Aiming to fill this gap, we have taken advantage of the recent publication of the *T. cruzi*, *T. brucei*, *Leishmania major, L. infantum* and *L. braziliensis* genomes. In a BLASTp search, using as query the available CfKAP protein sequences, and eliminating sequences that were redundant or without the characteristic kinetoplast amino terminus, we have identified 35 protein sequences related to CfKAPs (11 in *T. cruzi;* 7 in *L. braziliensis;* 6 in *L. major* and *L. infantum;* and 5 in *T. brucei*) (Table 1). A phylogenetic analysis including these 35 sequences and the five CfKAPs used as query was performed, in order to construct a phylogenetic tree. The homology identification by sequence analysis is, for some genes, of low confidence. This can be explained by the small size of KAPs and, mainly, by the lower information content on their amino acid composition, enriched for arginine, lysine and alanine, having few amino acid positions that are gene specific. In order to increase the information content for predicting homologous relationships, a synteny conservation analysis was performed (figures 1.1-1.3), where chromosome location was highly correlated with tree topology, allowing us to infer the homology relationships between the trypanosomatid KAPs. The KAP genes of *T. cruzi* are present as two copies, with the exception of TcKAP4c, probably due to the hybrid nature of the CL Brener strain. They were considered as alleles in the present analysis and numbered accordingly (for instance, TcKAP3.1 and TcKAP3.2).

The KAP1 and KAP2 genes were only identified in *Leishmania* spp. and *C. fasciculata* (figures 2 and 3). Regarding the KAP1 gene, the most similar sequences in *T. cruzi* and *T. brucei* were larger and lacked the amino terminal presequence, having been annotated as 60S ribosomal protein L19 and histone H1, respectively. The KAP2 gene is relatively similar to the KAP3 gene (figure 5), being located in the same genome region in tandem (figure 1.1), as previously reported.

The KAP3 and KAP4 genes were identified in all trypanosomatids analyzed. The TbKAP3 gene is considerably larger (36.6 kDa) when compared to the average predicted molecular weight for *T. cruzi, C. fasciculata* and *Leishmania* sp. (18.4 kDa), presenting a longer carboxyl terminal tail (figure 4). Regarding the KAP4 gene, in *Trypanosoma* sp., there is evidence of paralogy, as *T. brucei* has two copies of this gene and *T. cruzi* has three. *L. braziliensis* has also an extra copy of the KAP4 gene, but it is shorter (Table 1; figure 6) and in a distinct genome position, although juxtaposed to the prototype LbKAP4 (figure 1.2). The common two copies in *Trypanosoma* sp. are interspersed by another KAP related protein, KAP7, as described below. The KAP4 potential paralogs are clustered together, in a species-specific manner. Although it is possible that they have arisen, in *T. cruzi* and *T. brucei*, as two independent events, it is more parsimonious to propose a single duplication event, followed by an intra-species sequence homogenization driven by gene conversion, what is reinforced by the existence of KAP7 gene between KAP4a and KAP4b in both species.

As previously stated, originally five distinct proteins were described in *C. fasciculata*, named CfKAP1-5, which were directly or indirectly associated with kDNA. CfKAP5, also designated p15, was never characterized. Taking into account the phylogenetic and syntenic analysis made in this paper, we were able to identify two other genes that are similar to the CfKAPs, which have been tentatively named KAP6 and KAP7. They have not been characterized in *Crithidia*, as the available sequence information for this genome is limited (227 nucleotide sequences in the current version of GenBank).

The KAP6 gene (figure 1.3) has a size compatible to other KAPs (18.5 kDa in *Leishmania;* 22 kDa in *Trypanosoma*), an amino terminus similar to the characteristic presequence and an amino acid composition rich in lysine and arginine (figure 7). It is annotated in all five genome analyzed as “kinetoplast DNA-associated protein” and it is more related to KAP4. The KAP7 gene is also present in all trypanosomatids and has been annotated as “hypothetical protein, conserved”. Although KAP7 is larger than other KAP proteins (38 kDa in *Leishmania;* 25 kDa in *Trypanosoma*), it has several KAPs characteristics, such as an amino terminus similar to the characteristic presequence, the amino acid composition rich in lysine, arginine and alanine (figure 8), and hence, a basic predicted isoelectric point. It is located adjacent to the KAP4 gene in all genome analyzed (figure 1.2), having a slight higher similarity to this gene by BLASTp analysis. Although it is clustered with the KAP1 gene in the phylogenetic tree, the lower bootstrap value of this clade reinforces the uncertainty of KAP7 relationship to other KAPs.

Table 1. Trypanosomatid KAPs selected in the present work.

| **Gene Name** | **Genbank ID** | **MW** | **pI** |
| --- | --- | --- | --- |
| CfKap1 | AAC32801 | 15.0 | 12.7 |
| LbKap1 | XP_001568116 | 14.6 | 12.0 |
| LiKap1 | XP_001468384 | 14.1 | 12.3 |
| LmKap1 | XP_001686086 | 14.9 | 12.4 |
| CfKap2.1 | AAB70749 | 14.8 | 11.6 |
| CfKap2.2 | AAC47741 | 14.9 | 11.4 |
| LbKap2 | XP_001567749 | 15.2 | 11.7 |
| LiKap2 | XP_001468064 | 15.0 | 11.8 |
| LmKap2 | XP_001685703 | 14.9 | 11.7 |
| CfKap3 | AAN46297 | 15.3 | 11.0 |
| LbKap3 | XP_001567750 | 16.6 | 11.6 |
| LiKap3 | XP_001468065 | 16.0 | 11.7 |
| LmKap3 | XP_001685704 | 16.0 | 11.5 |
| TbKap3 | XP_829697 | 36.6 | 11.8 |
| TcKap3a | XP_803126 | 23.8 | 12.1 |
| TcKap3b | XP_815726 | 22.5 | 12.1 |
| CfKap4 | 2206467C | 14.6 | 10.7 |
| LbKap4 | XP_001569226 | 14.1 | 10.9 |
| LbKap4tr | XP_001569227 | 6.2 | 10.4 |
| LiKap4 | XP_001469966 | 14.1 | 11.2 |
| LmKap4 | XP_001687172 | 14.2 | 11.3 |
| TbKap4.1 | XP_823119 | 21.2 | 10.3 |
| TbKap4.2 | XP_823121 | 14.3 | 10.8 |
| TcKap4.1a | XP_808913 | 14.4 | 11.5 |
| TcKap4.1b | XP_807061 | 14.5 | 11.4 |
| TcKap4.2a | XP_808911 | 14.7 | 10.6 |
| TcKap4.2b | XP_807063 | 14.2 | 10.6 |
| TcKap4.3 | XP_807064 | 14.6 | 10.8 |
| LbKap6 | XP_001569234 | 18.9 | 11.6 |
| LiKap6 | XP_001469974 | 18.4 | 11.5 |
| LmKap6 | XP_001687180 | 18.3 | 11.3 |
| TbKap6 | XP_823113 | 23.5 | 10.6 |
| TcKap6a | XP_815942 | 21.2 | 11.3 |
| TcKap6b | XP_806801 | 21.3 | 11.3 |
| LbKap7 | XP_001569225 | 37.8 | 9.9 |
| LiKap7 | XP_001469965 | 37.9 | 10.2 |
| LmKap7 | XP_001687171 | 37.7 | 10.1 |
| TbKap7 | XP_823120 | 26.9 | 10.9 |
| TcKap7a | XP_808912 | 22.8 | 11.2 |
| TcKap7b | XP_807062 | 38.5 | 10.2 |

**Gene Name:** Nomenclature attributed, based on the original names of CfKAPs and the phylogenetic analysis.

**GenBank ID:** Accession ID of the protein sequence used in the evolutionary analysis.

**MW:** Molecular weight.

**pI:** Isoelectric point


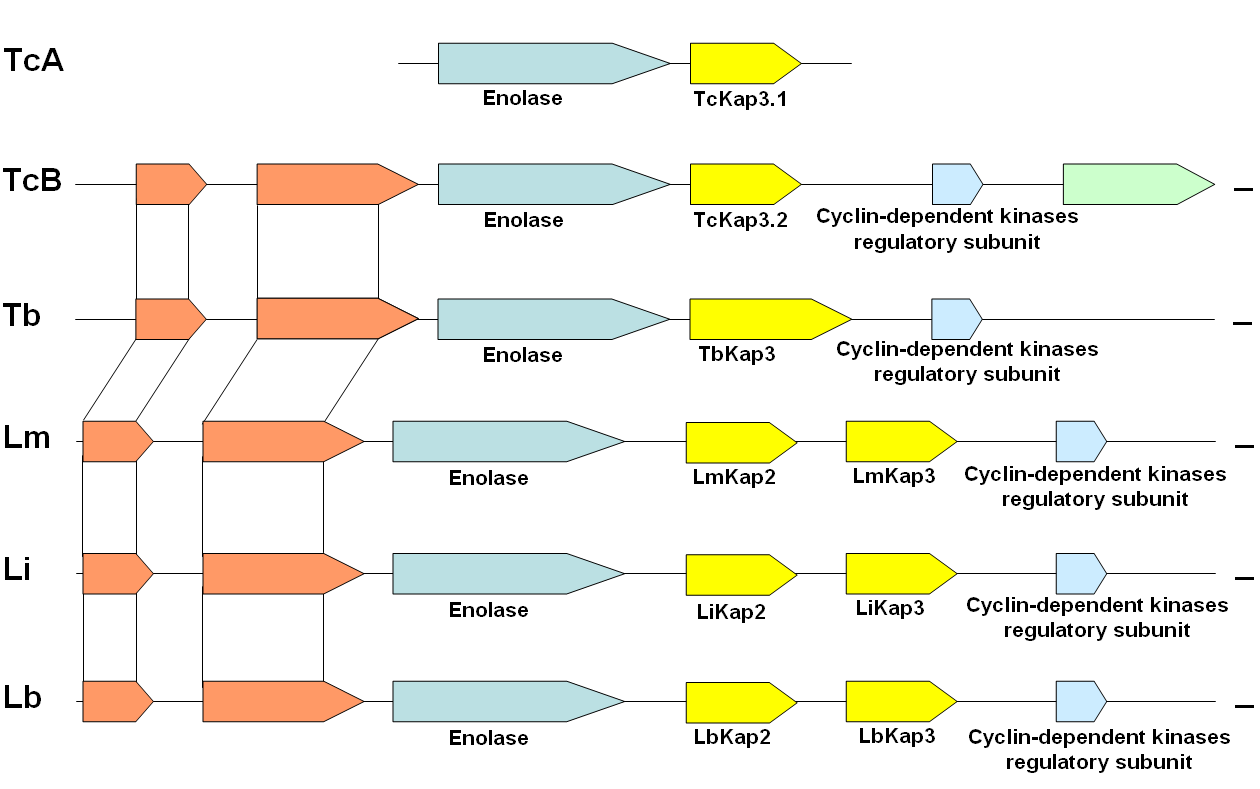
Figure 1.1. KAP2 and KAP3 genome location in the five genomes analyzed (see notes below)


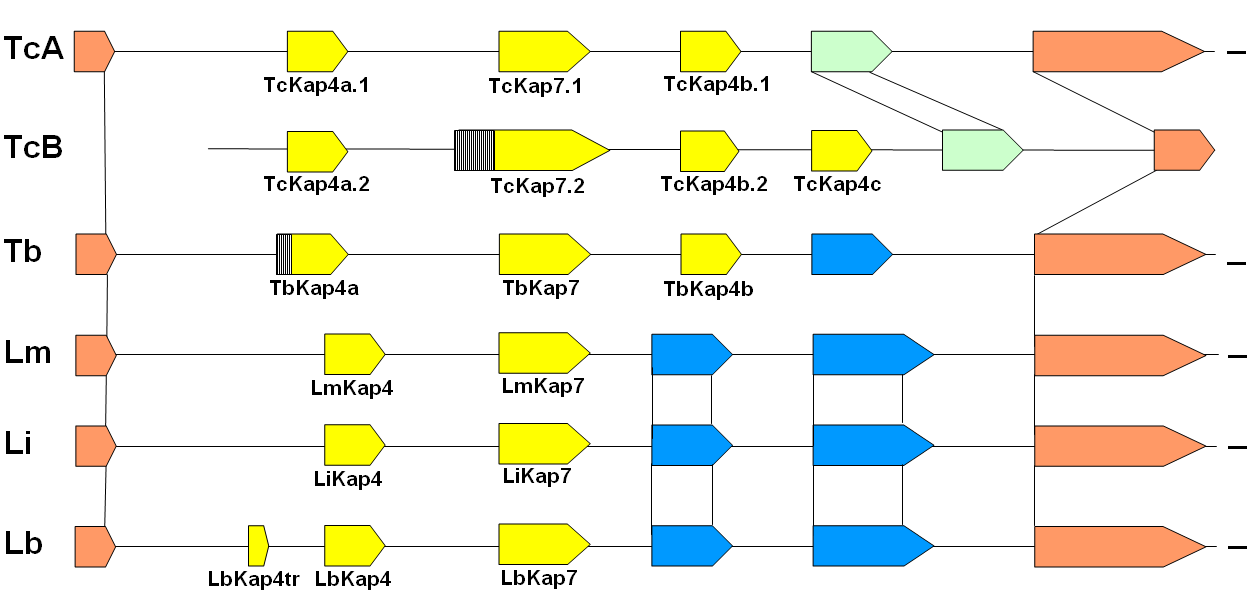


Figure 1.2. KAP4 and KAP7 genome location in the five genomes analyzed (see notes below)


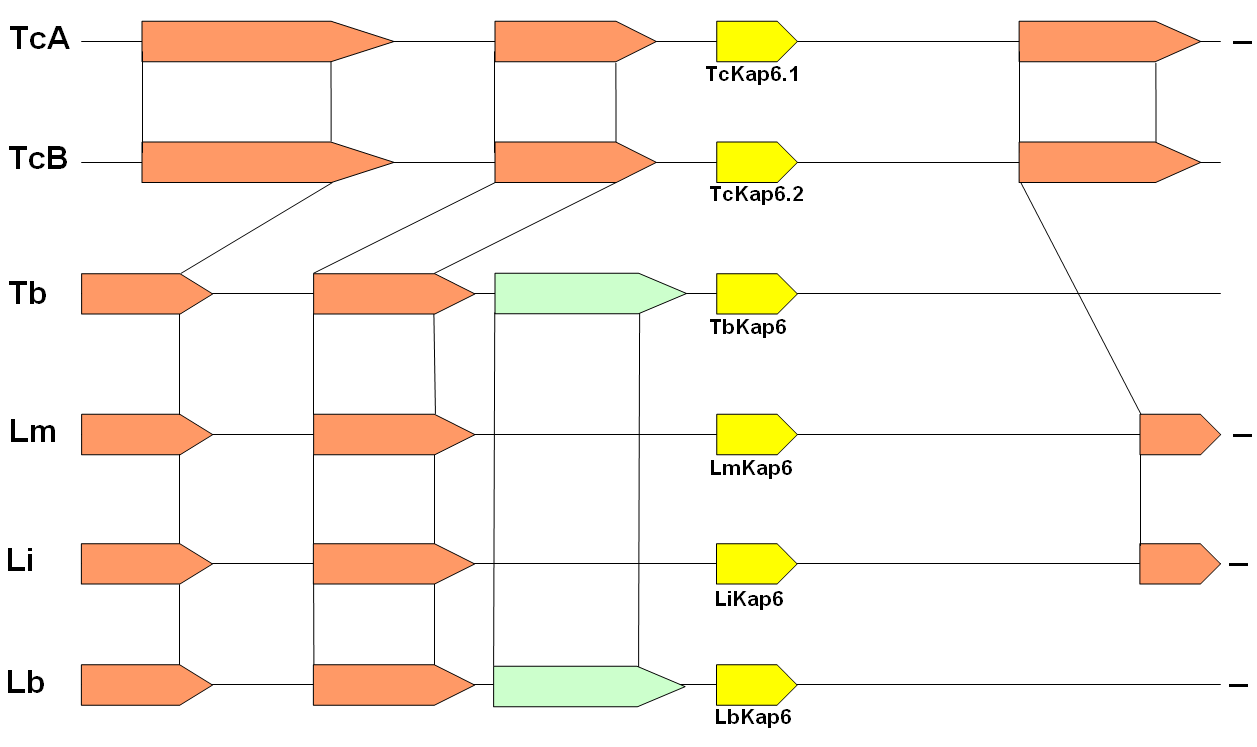
Figure 1.3. KAP6 genome location in the five genomes analyzed (see notes below)

Notes on figures 1.

Gene size and position are depicted in an approximate form, with emphasis on graphical clarity.

The KAP genes are colored in yellow.

Genes with an annotation more informative on function are colored in light blue.

Genes annotated as “hypothetical conserved” are colored in orange.

Genes annotated as “hypothetical” are colored in green.

Genes that has no synteny conservation in relation to *T. cruzi* are colored in blue.

Genes in yellow and light blue do not have lines representing the orthology relationship as this can be deduced from their names/annotation. The other genes have vertical lines connecting them, which represents their orthologous relationship.

All genome fragments are organized, from left to right, to be in the translation sense. If, in order to do so, we had to inverse the genome fragment direction, a minus sign is placed in the rightmost part of the figure.

All information is exactly as provided by the genome sequencing consortia. No attempt was made to modify/verify gene identification, with the exception of a possible error in the definition of the CDS starting point, as shown for TbKAP4a and TcKAP7.2.


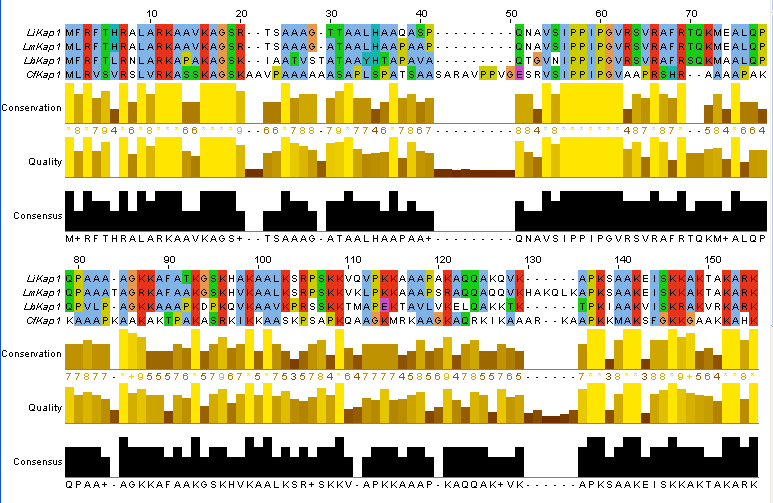


Figure 2. Alignment of the KAP1 gene, colored accordingly to the ClustalW pallete.


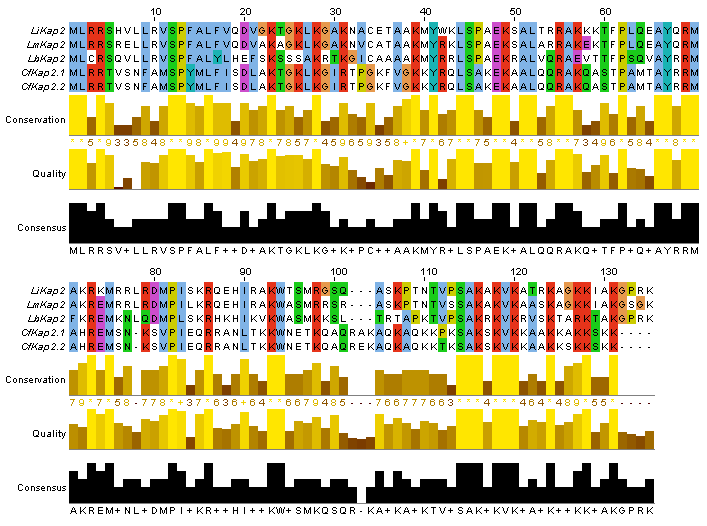


Figure 3. Alignment of the KAP2 gene, colored accordingly to the ClustalW pallete.


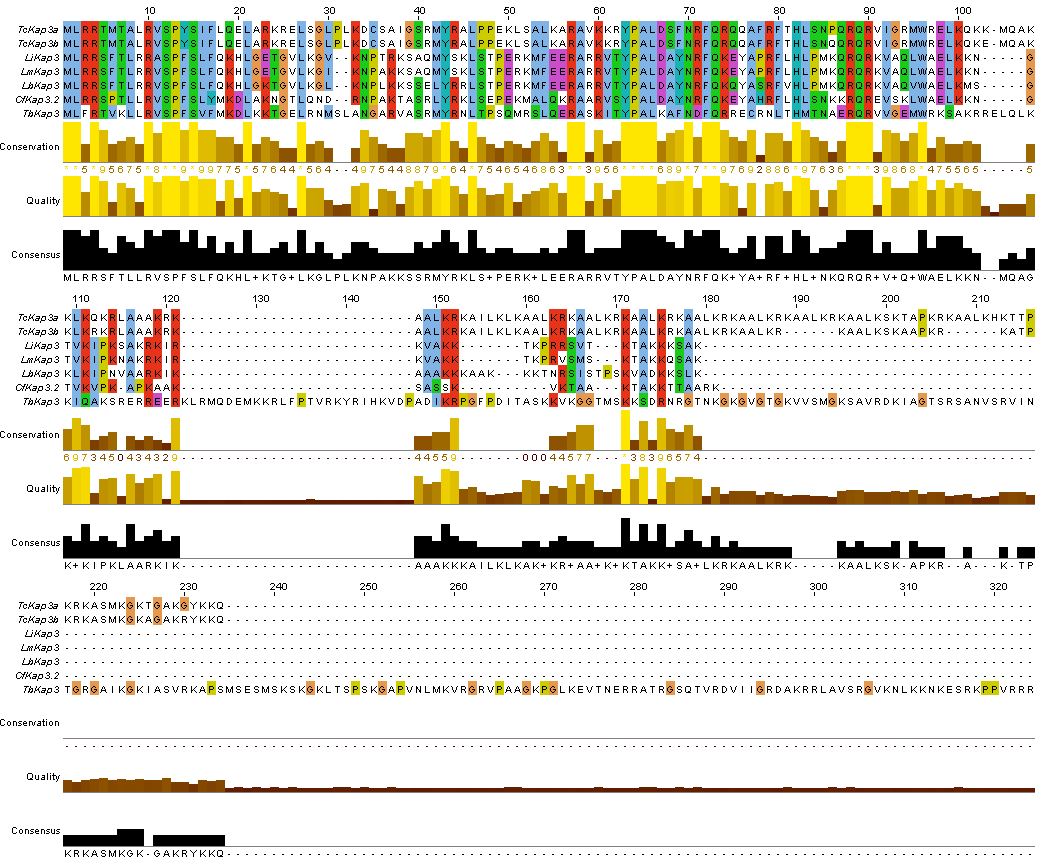


Figure 4. Alignment of the KAP3 gene, colored accordingly to the ClustalW pallete.


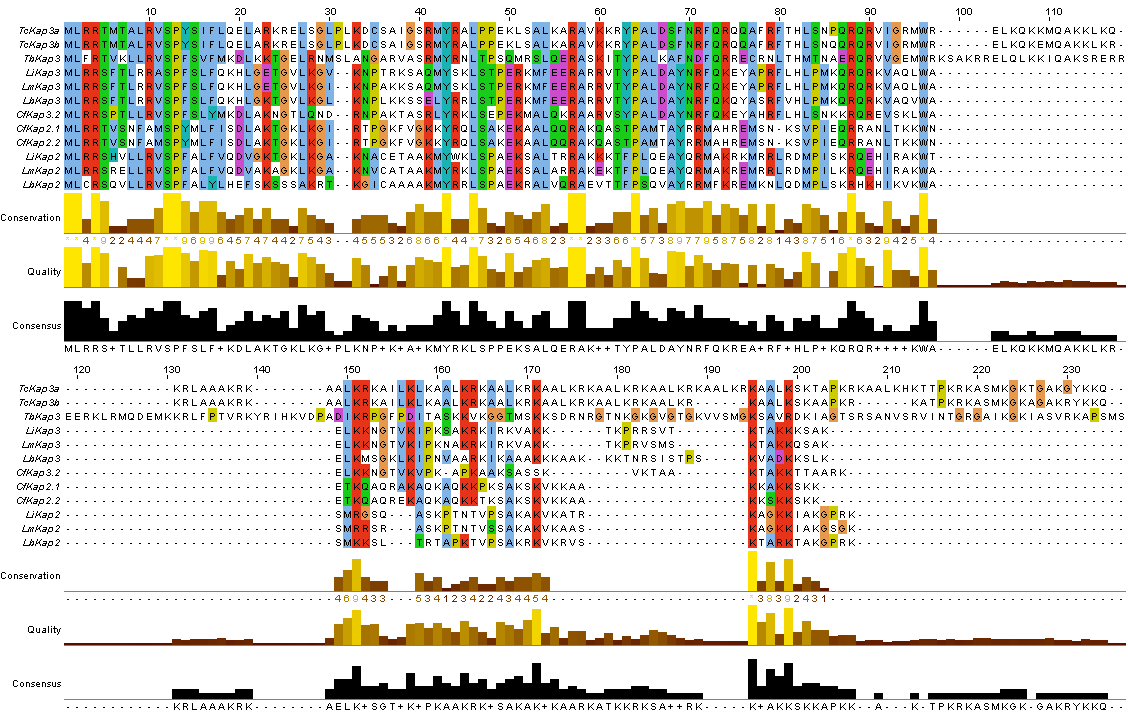


Figure 5. Alignment of the KAP2 and KAP3 gene, colored accordingly to the ClustalW pallete.


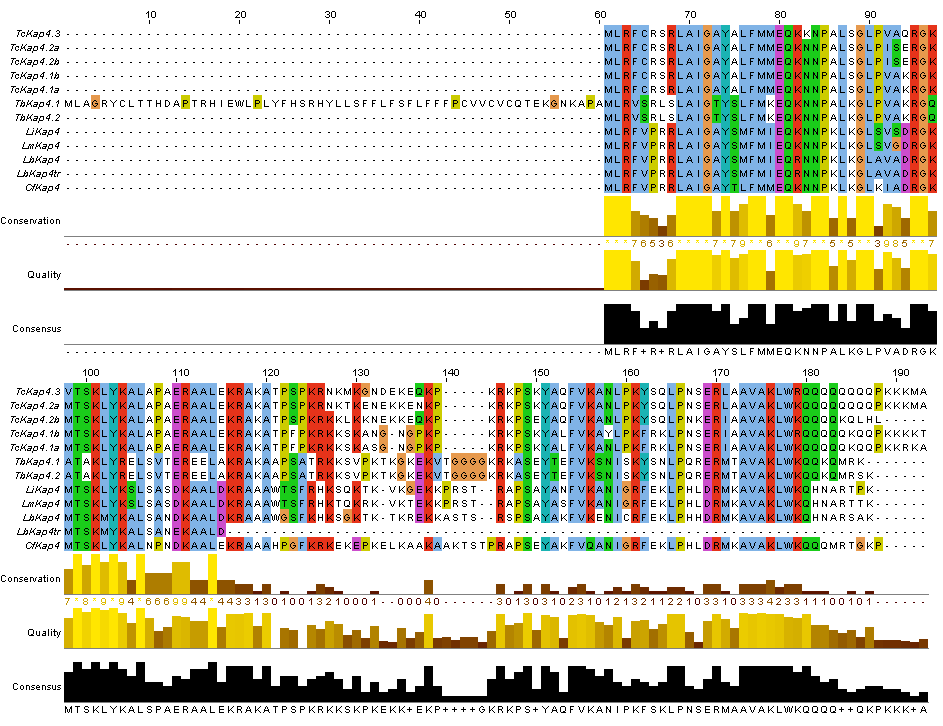


Figure 6. Alignment of the KAP4 gene, colored accordingly to the ClustalW pallete.


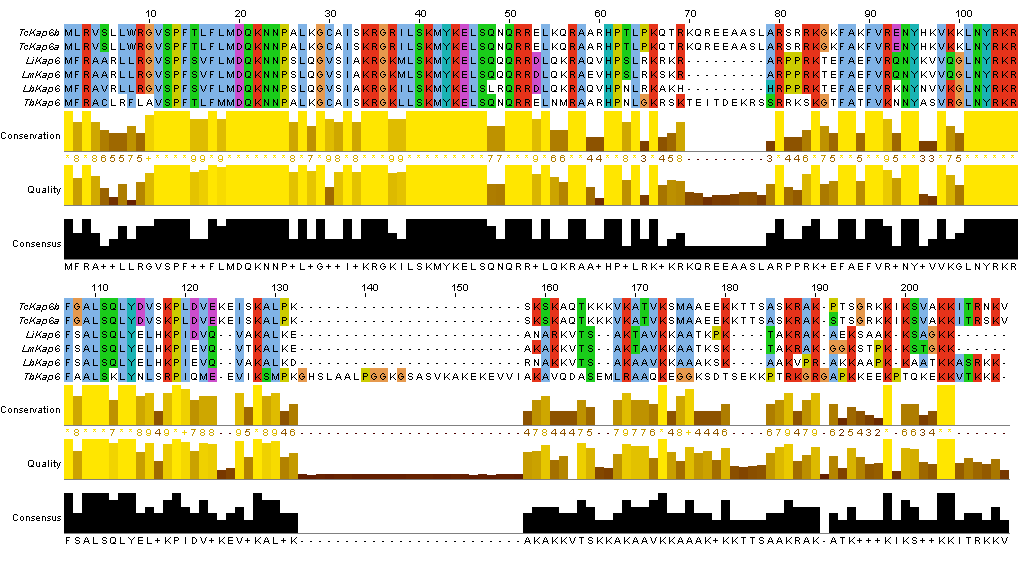
Figure 7. Alignment of the KAP6 gene, colored accordingly to the ClustalW pallete.


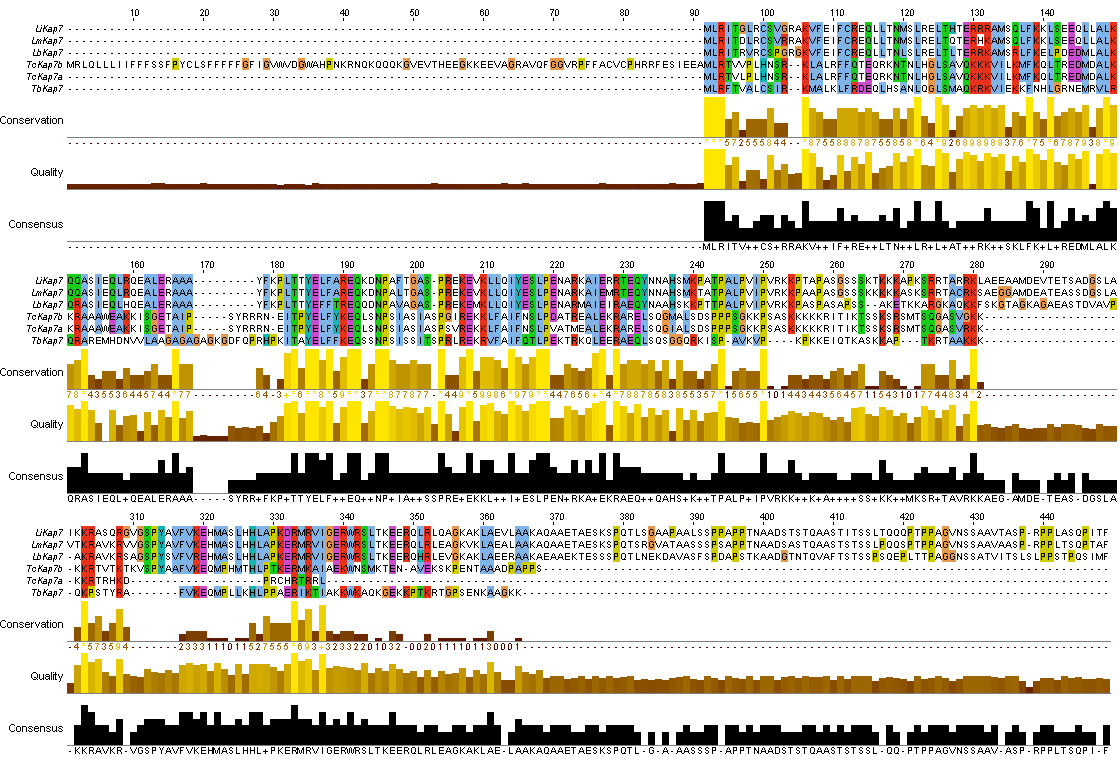


Figure 8. Alignment of the KAP7 gene, colored accordingly to the ClustalW pallete.
